# Supplementary material for: Sleep spindles track cortical learning patterns for memory consolidation
Source: Curr Biol. 2022 Jun 6;32(11):2349–2356.e4. doi: 10.1016/j.cub.2022.04.045 (PMC9616732; doi:10.1016/j.cub.2022.04.045)
Supplement: Document S1. Figures S1–S3 and Tables S1 and S2 [file mmc1.pdf]

Current Biology, Volume *32*

Supplemental Information

Sleep spindles track cortical learning patterns for memory consolidation

Marit Petzka, Alex Chatburn, Ian Charest, George M. Balanos, and Bernhard P. Staresina

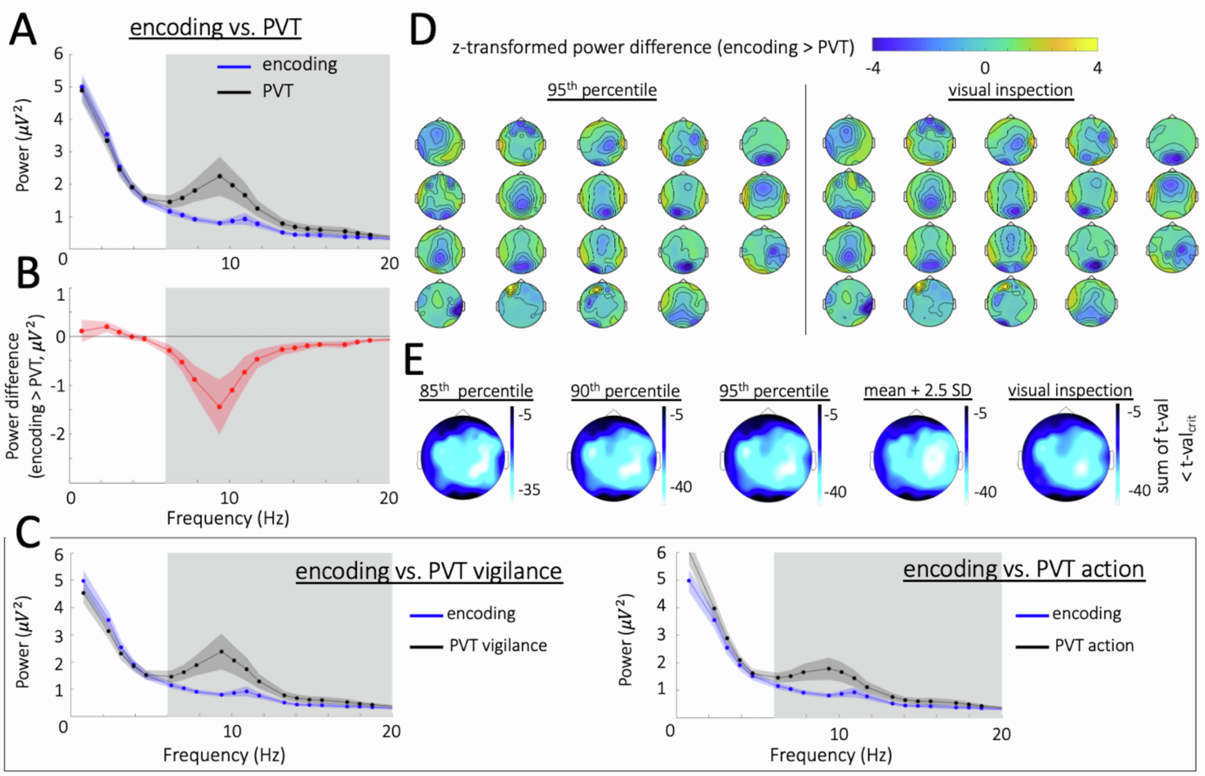

Figure S1. Frequency spectra and topographies of power differences between encoding and PVT. Related to Figure 1E.

(A) Power spectra (mean +/- SEM) for encoding (blue), PVT (black) as well as their difference (red, shown in B, statistically significant differences are highlighted in grey, $p < .05$ cluster corrected) are shown for electrode CP4 (cf., main Figure 1E). These were the raw values entering statistical analyses presented in main Figure 1E. (C) Power spectra (mean +/- SEM) for encoding and PVT 'vigilance' (left) as well as encoding and PVT 'action' (right). For the PVT 'vigilance' component, 10 sec pre-counter (minimum for which the fixation cross, without any motor responses, was presented) and for the PVT 'action', 4 seconds post-counter (time during which motor responses were given) were included. Statistically significant differences are highlighted in grey, $p < .05$ cluster corrected. (D) Participant-specific topographies of the 6-20 Hz power changes during encoding relative to the PVT (after excluding epochs based on the 95th percentile or after visual inspection). Power changes were z-transformed for comparability across participants. (E) Applying different thresholds (85th, 90th, 95th percentile, mean + 2.5 SD or visual inspection) to exclude potential artifacts result in comparable encoding patterns (the frequency range in which encoding and PVT significantly differ is always 6-20Hz).

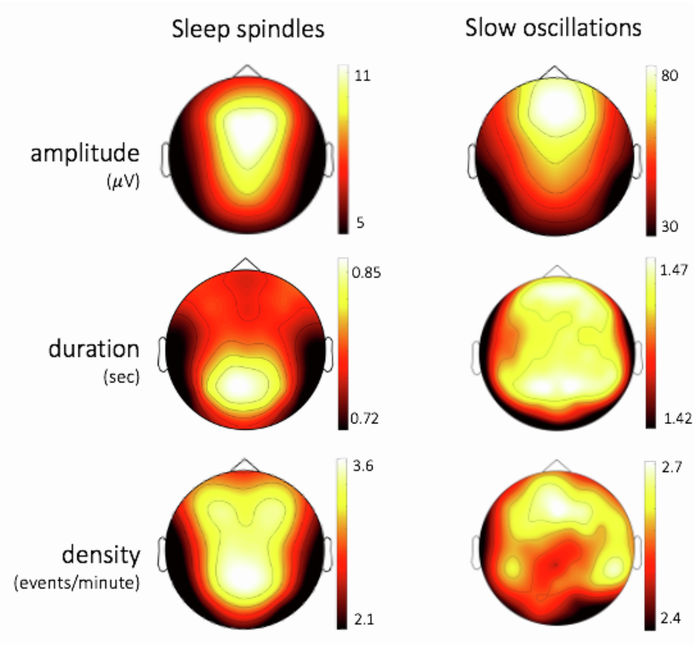

Figure S2. Group-level topographies of amplitude, duration and density of sleep spindles and slow oscillations. Related to Figure 2A.

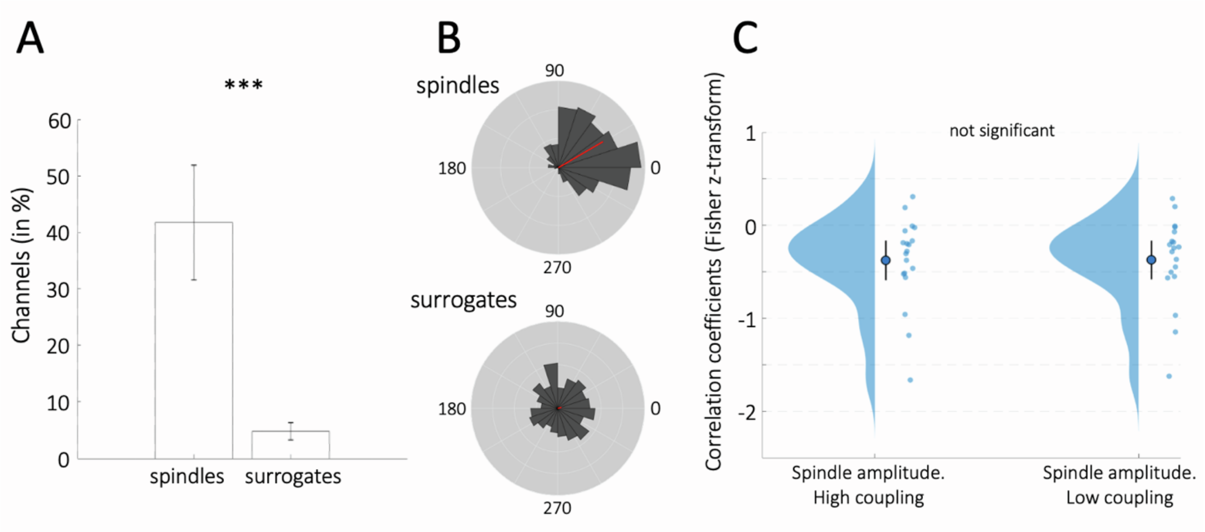

Figure S3. Coupling of spindles and surrogate events to the phase of the signal filtered in the SO frequency range (0.3 -1.25). Related to Figure 2B.

(A) Mean (+/- 95% confidence intervals) percentage of channels on which sleep spindles and surrogate events are significantly coupled (defined by a significant deviation from a uniform distribution, Rayleigh test: $p < .05$). Spindles are coupled to SOs on significantly more channels than surrogates. Surrogates were matched control events - for each detected spindle, a spindle-free epoch within 15 seconds before or after the actual spindle event was identified [S1]. The instantaneous phase angle of the SO filtered and Hilbert transformed signal was then extracted at the centre of the spindle-free epoch.

(B) The corresponding phase (in degrees) of spindle maxima (top) and surrogate centres (bottom) plotted across all channels with significant spindle coupling (including all participants, fixed-effects, $n = 447$). While spindles significantly cluster at a phase of 30 degrees (Rayleigh test: $z = 173.75$, $p < .001$, resultant vector length = 0.62), surrogates deviate only moderately from a uniform distribution (Rayleigh test: $z = 3.22$, $p = .040$, resultant vector length = 0.08). Both distributions significantly differ (Kuiper test: $p < .001$).

(C) No differential encoding-spindle amplitude overlap for spindles with higher (left) vs. lower (right) coupling to the SO up-state.

	N1	N2	N3	REM	TST
mean	18.39	52.53	10.42	16.24	103.58
SEM	2.46	3.66	2.56	2.85	2.28

Table S1. Descriptive sleep data in minutes (mean and SEM). Related to Figure 1C and 2A.
n = 19. TST = total sleep time.

**electrode Fz**	**sleep spindles**			**slow oscillations**		
	amplitude	duration	density	amplitude	duration	density
mean	11.13	0.76	3.27	82.97	1.42	2.49
SEM	0.63	0.01	0.16	6.11	0.01	0.13
**electrode Cz**						
mean	11.03	0.80	3.45	68.07	1.41	2.67
SEM	0.54	0.01	0.19	4.49	0.01	0.12
**all electrodes**						
mean	7.83	0.78	3.02	56.01	1.42	2.62
sem	0.36	0.01	0.15	4.14	0.01	0.12

Table S2. Descriptive data of sleep oscillations. Related to Figure 2A.
Descriptive data of amplitude (maximum peak for spindles and absolute value of the most negative trough for slow oscillations, in μV), duration (time from beginning to end of algorithmically detected events, in seconds) and density (n events/minute) of sleep spindles and slow oscillations for electrode position Fz, Cz and averaged across all electrodes (mean and SEM). n = 19.

Supplemental References

S1. Ngo, H.V. V., Fell, J., and Staresina, B. (2020). Sleep spindles mediate hippocampal-neocortical coupling during long-duration ripples. Elife *9*, 1–18.
