# Supplementary material for: Sleep spindles track cortical learning patterns for memory consolidation
Source: Curr Biol. 2022 Jun 6;32(11):2349–2356.e4. doi: 10.1016/j.cub.2022.04.045 (PMC9616732; doi:10.1016/j.cub.2022.04.045)
Supplement: Document S2. Article plus supplemental information [file mmc2.pdf]

Current Biology

Article

Sleep spindles track cortical learning patterns for memory consolidation

Highlights

- Participant-specific topographies of sleep spindles overlap with learning topographies
- This overlap is driven by sleep spindle amplitudes
- The overlap of learning/spindle topographies predicts memory consolidation

Authors

Marit Petzka, Alex Chatburn, Ian Charest, George M. Balanos, Bernhard P. Staresina

Correspondence

bernhard.staresina@psy.ox.ac.uk

In brief

Petzka et al. provide evidence that post-learning sleep spindles track learning-related cortical networks and thereby facilitate memory consolidation.

Petzka et al., 2022, Current Biology *32*, 2349–2356
June 6, 2022 © 2022 The Author(s). Published by Elsevier Inc.
https://doi.org/10.1016/j.cub.2022.04.045

CellPress

Current Biology

CellPress
OPEN ACCESS

Article

Sleep spindles track cortical learning patterns for memory consolidation

Marit Petzka,[1,2] Alex Chatburn,[3] Ian Charest,[4] George M. Balanos,[5] and Bernhard P. Staresina[6,7,8,*]
[1]School of Psychology and Centre for Human Brain Health, University of Birmingham, Birmingham, UK
[2]Max Planck Research Group NeuroCode, Max Planck Institute for Human Development, Berlin, Germany
[3]Cognitive and Systems Neuroscience Research Hub, University of South Australia, Adelaide, SA, Australia
[4]Department of Psychology, University of Montreal, Montreal, QC, Canada
[5]School of Sport, Exercise and Rehabilitation, University of Birmingham, Birmingham, UK
[6]Department of Experimental Psychology, University of Oxford, Oxford, UK
[7]Oxford Centre for Human Brain Activity, Wellcome Centre for Integrative Neuroimaging, Department of Psychiatry, University of Oxford, Oxford, UK
[8]Lead contact
*Correspondence: bernhard.staresina@psy.ox.ac.uk
https://doi.org/10.1016/j.cub.2022.04.045

SUMMARY

Memory consolidation—the transformation of labile memory traces into stable long-term representations—is facilitated by post-learning sleep. Computational and biophysical models suggest that sleep spindles may play a key mechanistic role for consolidation, igniting structural changes at cortical sites involved in prior learning. Here, we tested the resulting prediction that spindles are most pronounced over learning-related cortical areas and that the extent of this learning-spindle overlap predicts behavioral measures of memory consolidation. Using high-density scalp electroencephalography (EEG) and polysomnography (PSG) in healthy volunteers, we first identified cortical areas engaged during a temporospatial associative memory task (power decreases in the alpha/beta frequency range, 6–20 Hz). Critically, we found that participant-specific topographies (i.e., spatial distributions) of post-learning sleep spindle amplitude correlated with participant-specific learning topographies. Importantly, the extent to which spindles tracked learning patterns further predicted memory consolidation across participants. Our results provide empirical evidence for a role of post-learning sleep spindles in tracking learning networks, thereby facilitating memory consolidation.

INTRODUCTION

Sleep after learning bolsters memory retention, a process referred to as sleep-dependent memory consolidation.[1–3] In recent years, sleep spindles—transient 12–15 Hz oscillations generated within thalamocortical loops—have emerged as a prime mechanistic vehicle to support consolidation.[4–9] Previous studies have linked spindles to consolidation in terms of their density (the number of discrete spindle events per minute),[10] power,[11] and activity, a combination of duration and amplitude.[12] Despite their ubiquity, however, the specific role that spindles play for memory consolidation remains poorly understood.

Ultimately, effective learning requires structural brain changes, beginning at the synaptic level.[13,14] A hallmark computational/biophysical framework[15] suggests that spindles are particularly well-suited to induce changes in synaptic plasticity. Specifically, spindles gate influx of calcium (Ca^{2+}) into pyramidal dendrites, setting early synaptic consolidation processes in motion. Empirical support for this model has been provided by the *in vitro* application of spindle-like firing patterns,[16] as well as by showing a direct modulation of Ca^{2+} activity in cortical pyramidal dendrites as a function of spindle power during natural sleep in rodents.[17] Moreover, cortical microelectrode array recordings in humans have shown that spindles group co-firing of single units within 25 ms, i.e., within a time window conducive to spike-timing-dependent plasticity.[18] Critically, however, for spindles to promote memory consolidation in an adaptive fashion, they need to show some degree of regional specificity. That is, not only would global synaptic consolidation be of limited use in an ever-changing landscape of tasks, but it would also be at direct odds with extant models emphasizing the role of sleep in global synaptic downscaling.[19] Instead, adaptive consolidation has to be selective, specifically strengthening local circuits involved in prior learning.

The current study thus set out to assess (1) whether spindles track specific pre-sleep learning patterns and (2) whether this learning-spindle overlap supports memory consolidation. To this end, we employed a demanding memory task giving rise to rich and idiosyncratic activation patterns during encoding (Figure 1 [Memory Arena]). After learning, participants took a 2-h nap before their memory retention was tested. This protocol allowed us to examine whether spindles recorded during this nap would track participant-specific learning patterns and whether the extent of this learning-sleep overlap would predict behavioral expressions of consolidation.

© 2022 The Author(s). Published by Elsevier Inc.
This is an open access article under the CC BY license (http://creativecommons.org/licenses/by/4.0/).

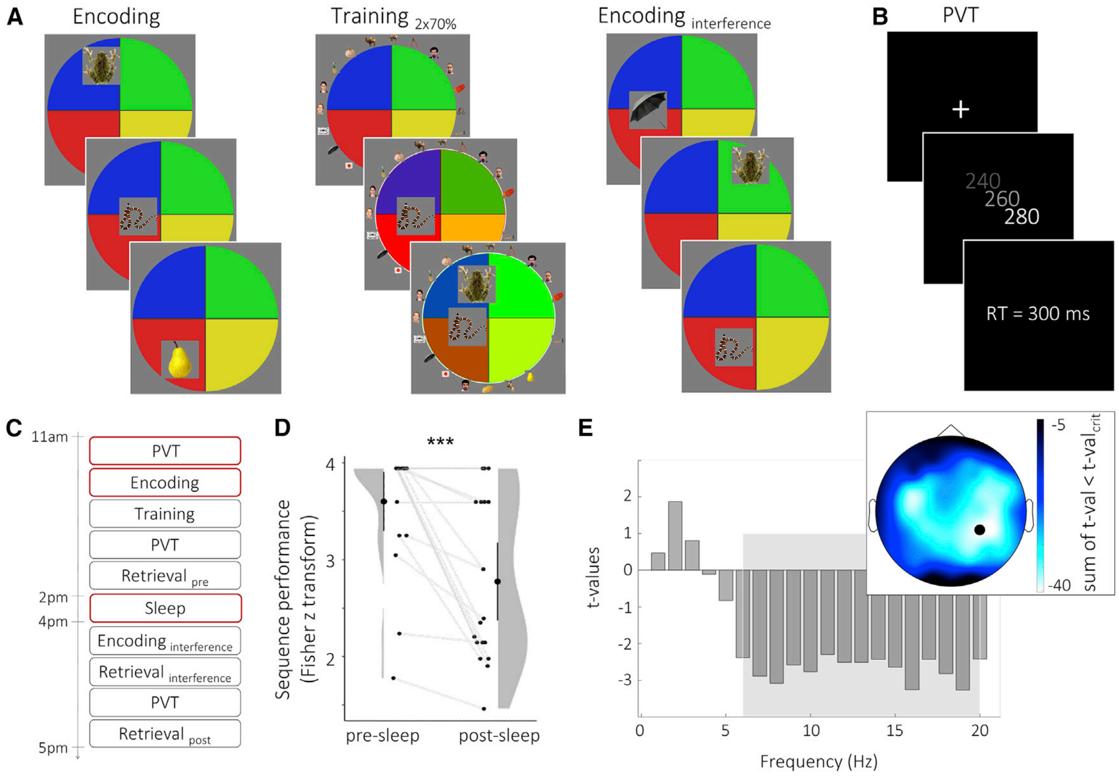

Figure 1. Task, experimental design, behavioral results, and group EEG encoding pattern

(A) Memory Arena. During encoding, 20 objects were presented in a specific sequence at different spatial positions. Both sequence and spatial position had to be encoded. Training (and retrieval) started with all 20 objects arranged around the arena, and participants had to drag and drop the objects in the correct sequence to the correct spatial position. During training, feedback was given after each trial and errors were corrected. Training was completed after reaching a performance criterion of 70% twice in a row. Retroactive interference was induced by encoding the same objects but in a different sequence and at different spatial positions.

(B) Every trial of the psychomotor vigilance task (PVT) started with a fixation cross. After a delay of 10–15 s, a counter started. Participants had to press the space bar as fast as possible and received feedback about their reaction time (RT).

(C) Participants performed the PVT, encoding, training, and first retrieval before the 2-h nap. After the nap, an interference session was employed (1× encoding and retrieval, no training), followed by the second retrieval of the originally learned arena. For EEG data analyses, the first PVT, encoding, and sleep data were used.

(D) Sequence memory significantly decreased from pre- to post-sleep. Single participant data, density plots, and group means with 95% CIs are shown. ***$p < 0.001$.

(E) Comparison of oscillatory power during Memory Arena versus PVT (thresholded at $p < 0.05$ cluster corrected), revealing a significant power decrease from 6–20 Hz during encoding (gray rectangle), most pronounced over temporo-parietal areas (bars shown for electrode CP4, black circle on topography plot; see Figures S1A and S1B for raw values).

RESULTS

Behavioral results

We employed a recently developed memory paradigm called "Memory Arena,[20]" in which participants learn the temporospatial arrangement of objects in a circular enclosure across multiple training rounds (see Figure 1A and STAR Methods for details). Memory for the temporospatial arrangement was assessed in a first retrieval block (pre-sleep retrieval), which was followed by a 2-h nap (see Table S1 for descriptive data of sleep stages). In our previous work, we found that the sensitivity to detect sleep-dependent consolidation effects benefits from the inclusion of a retroactive interference task, particularly when pre-sleep memory performance is relatively high.[20] We thus also included a retroactive interference task in the current paradigm. Specifically, participants were instructed to learn a new temporospatial arrangement of the same objects (retroactive interference), after which they were asked to retrieve the original arrangement (post-sleep retrieval; Figure 1C). As the dependent variable, we use sequence memory performance, as our previous work suggested this was the measure most sensitive to capture sleep-dependent consolidation. As expected, we found a significant decrease in sequence performance from pre- to post-sleep retrieval of the original sequence ($t(18) = 4.65$, $p < 0.001$, $d = 1.07$; Figure 1D). For further analyses, memory consolidation is defined as memory retention, i.e., the relative change in sequence performance from pre- to post-sleep retrieval.

EEG results: Spindle amplitude tracks encoding patterns

To assess whether sleep spindles track learning sites, we first derived an "encoding pattern" for each participant. To

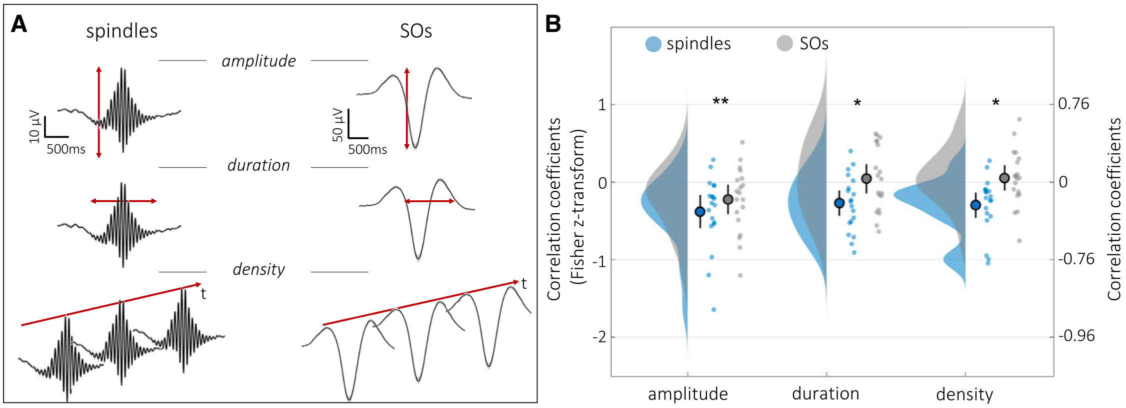

Figure 2. Sleep spindles track cortical learning sites

(A) Grand average (mean ± 95% CIs) of spindles and SOs at electrode position Cz. Amplitude, duration, and density were extracted for each participant and channel (see Table S2 for corresponding descriptive data and Figure S2 for group-level topographies).

(B) Across participants, encoding topographies were significantly more strongly correlated with topographies of spindles than SOs (density plots, group means, 95% CIs, and single participant data are shown. **p < 0.01. *p < 0.05).

specifically unravel learning-related activity, we contrasted oscillatory power (1–20 Hz, 1 s epochs with 50% overlap, Hanning-windowed) during encoding with power during a control condition (PVT, psychomotor vigilance task). On the group level, this contrast revealed a significant power decrease in the alpha/beta frequency range (6–20 Hz) during encoding relative to the PVT, particularly over right temporo-parietal areas (Figure 1E). The same power decreases in the alpha/beta frequency range were observed when contrasting encoding with different components of the PVT (i.e., vigilance and motor response; Figure S1C). This result is consistent with previous findings linking decreases in alpha/beta power to memory processes.[21–25] The reliable group effect notwithstanding, there was considerable variability in participant-specific effect topographies of the 6–20 Hz power decrease (Figure S1D), allowing us to explore whether these participant-specific encoding patterns would bias particular event characteristics during subsequent sleep.

As outlined in the introduction, we hypothesized that the topography (i.e., spatial distribution) of sleep spindles might be modulated by engagement during pre-sleep learning. We thus algorithmically detected sleep spindles during the post-learning nap (see STAR Methods) for every channel and extracted their amplitude as well as duration and density. As a control, we performed the same analyses for algorithmically detected slow oscillations (SOs), which have also been linked to memory consolidation.[11,26,27] At the group level, spindles and SOs showed the established prevalence over centro-parietal and frontal areas, respectively (see Figure S2 for amplitude, density, and duration topographies of spindles and SOs and Table S2 for descriptive data). Furthermore, and in line with previous observations,[28–31] detected spindle events were on average temporally coupled to the up-state of the SO signal (0.3–1.25 Hz, see Figures S3A and S3B).

We next turned to the question of whether inter-individual differences in the topography of sleep events relate to inter-individual differences in learning topographies. For each participant, the encoding topography (6–20 Hz power relative to the PVT across 58 channels) was correlated (Spearman's rho) with the corresponding topography of six different sleep patterns (amplitude, duration, and density for spindles and SOs across 58 channels). Note that all sleep measures are positively scaled, except the SO amplitude. For simplicity, we unified all scales by taking the absolute value of the SO amplitude. As encoding activity is associated with a decrease in power (negatively scaled values), encoding-sleep overlap would be signified by negative correlations.

Participant-specific correlation values were then evaluated at the group level. First, we conducted a 2 (event type: spindles versus SOs) × 3 (event characteristic: amplitude, duration, density) repeated measures ANOVA (Figure 2A), assessing whether particular sleep-event topographies track idiosyncratic encoding topographies. Results revealed that topographies of overall spindle characteristics correlated with encoding patterns to a greater extent than SO characteristics (main effect of event type $F(1,18) = 13.12$, $p < 0.001$, $\eta_p^2 = 0.11$; Figure 2B). This was the case for event amplitudes ($t(18) = -3.16$, $p_{corr} = 0.016$, $d = -0.72$) and durations ($t(18) = -2.53$, $p_{corr} = 0.042$, $d = -0.58$) as well as densities ($t(18) = -2.14$, $p_{corr} = 0.046$, $d = -0.49$). Furthermore, the correlation between all three spindle characteristics with the encoding pattern was significantly smaller than 0 (spindle amplitude: $mean_r = -0.38$, $t(18) = -3.50$, $p_{corr} = 0.015$, $d = 0.80$; spindle duration: $mean_r = -0.27$, $t(18) = -3.26$, $p_{corr} = 0.016$, $d = 0.75$; spindle density: $mean_r = -0.30$, $t(18) = -3.56$, $p_{corr} = 0.012$, $d = 0.82$). There was a trend for the main effect of event characteristic (amplitude topographies correlating strongest with encoding patterns, $F(2,36) = 2.61$, $p = 0.078$, $\eta_p^2 = 0.05$) and no interaction ($F(2,36) = 0.63$, $p = 0.532$, $\eta_p^2 = 0.01$).

To account for the possibility that spindle amplitude, duration, and density topographies are correlated among each other, we conducted follow-up partial correlations between encoding patterns and spindle characteristics. Interestingly, the link between encoding and spindle amplitude remained significantly different from 0 when partialling out spindle duration or spindle density (for both: $mean_r < -0.23$, $t(18) < -2.11$, $p < 0.049$, $d > 0.05$).

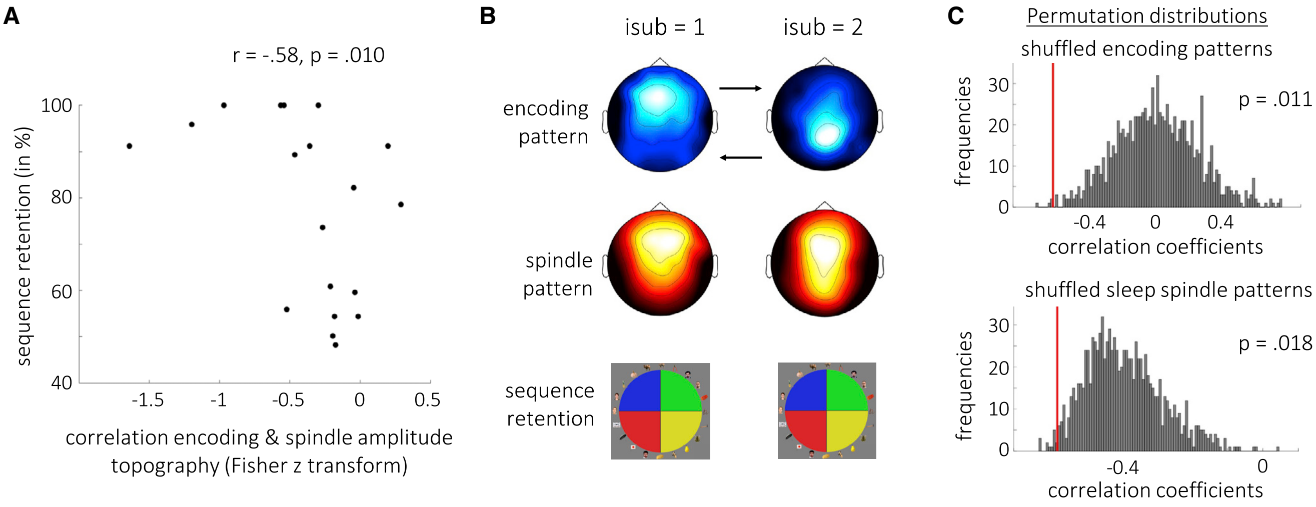

Figure 3. Extent of encoding-spindle overlap predicts memory consolidation

(A) The correlation between encoding and spindle amplitude topographies is predictive of sequence retention ($p < 0.05$). The more negative the correlation between spindle amplitude and encoding topographies (i.e., the greater the overlap), the greater the levels of sequence retention across sleep.

(B) Schematic for generating the null distributions in (C). Encoding or sleep spindle topographies were shuffled across participants, while the respective other topography (spindle amplitude or encoding power) and behavioral performance were retained.

(C) The observed correlation between encoding-spindle amplitude overlap and sequence retention exceeds both null distributions of encoding or spindle pattern exchangeability ($p < 0.05$).

When partialling out spindle amplitude, however, the overlap between spindle duration/density and encoding pattern was no longer significantly different from 0 (duration: $mean_r = -0.15$, $t(18) = -1.82$, $p = 0.086$, $d = 0.42$; density: $mean_r = -0.01$, $t(18) = -0.15$, $p = 0.885$, $d = 0.03$). Together, these results suggest that the spindle-encoding overlap is predominantly driven by spindle amplitude.

To examine whether the overlap with encoding patterns might be restricted to spindles that are coupled to SO up-states, we directly compared the encoding-spindle overlap (amplitude topographies) for spindle events with higher versus lower coupling (see STAR Methods). We observed no significant difference between the two event types ($t(18) = -0.66$, $p = 0.521$, $d = 0.33$; see Figure S3C).

Finally, we tested whether the overlap of sleep spindle amplitude and encoding activation is linked to behavioral expressions of memory consolidation. To this end, we correlated the encoding-spindle amplitude overlap with the relative change in sequence performance from pre- to post-sleep retrieval (memory retention) across participants. Indeed, a significant negative correlation was observed ($r = -0.58$, $p = 0.010$), indicating that participants who showed greater retention of sequence memory also had a greater overlap (signified by a more negative value) between encoding and sleep spindle topography (Figure 3A). To ensure that the link with behavior was driven by participant-specific encoding-spindle overlap, we first shuffled the encoding topographies between participants while retaining participant-specific sleep spindle topography and behavioral performance. Likewise, we shuffled the sleep spindle topographies between participants while retaining participant-specific encoding topography and behavioral performance (see Figure 3B for visualization). That way, we generated a distribution under the null hypothesis that (1) the encoding topography or (2) sleep spindle topography is irrelevant for the observed correlation with behavior. As shown in Figure 3C, the empirical correlation between encoding-spindle overlap and behavior significantly exceeded the null distribution in both cases ($p = 0.011$ for [1] and $p = 0.018$ for [2] based on 1,000 permutations). It is worth noting that the correlation between encoding-spindle overlap and behavior was specifically observed for the spindle amplitude (irrespective of higher versus lower coupling to SOs, both $r < -0.55$, $p < 0.016$). Neither the overlap between encoding and spindle duration nor that with spindle density correlated significantly with memory retention (for both: $r > -0.29$, $p > 0.226$).

DISCUSSION

Despite accumulating evidence linking sleep spindles to memory consolidation,[5–8,10–12,32] the specific function of spindles has remained elusive. Here, we tested predictions derived from biophysical/computational models, i.e., that spindles are preferentially expressed over learning-related sites where they might induce early stages of synaptic plasticity.[15] Using a recently developed paradigm sensitive to sleep-dependent memory consolidation[20] and geared toward eliciting rich idiosyncratic encoding patterns (Figure 1), we first demonstrate that sleep spindles—in particular, the topography of spindle amplitudes—track cortical patterns of memory encoding (Figure 2). This overlap of encoding patterns with spindles was significantly stronger than that with SOs, ruling out spurious correlations driven by generic signal properties across electroencephalography (EEG) channels. Importantly, we additionally reveal a functional link between the observed encoding-spindle overlap and memory consolidation, expressed in greater overlap being associated with greater levels of memory retention (Figure 3).

CellPress
OPEN ACCESS

Previous work has revealed a number of spindle characteristics that render them well-suited for inducing local plasticity in task-dependent/learning-related brain regions. For instance, intracranial recordings in humans have shown that sleep spindles are local rather than global,[33,34] and scalp EEG/magnetoencephalography (MEG) has demonstrated high levels of inter-subject variability of spindle topographies.[32,35] Moreover, spindle topographies vary as a function of prior learning tasks at the group level—spindles over left frontal areas are related to the consolidation of verbal material,[36] whereas spindles over parietal areas are related to the consolidation of visuospatial memories.[37] However, as brain activity was not measured during learning in these studies, the link between learning activation and post-learning spindle topography remained conjectural. Another study employed simultaneous EEG-fMRI recordings and found a blood-oxygen-level-dependent (BOLD) increase in learning-related ventro-temporal regions time-locked to sleep spindles at electrode position Cz,[38] but whether spindles per se would be preferentially expressed at learning-related sites remained unclear due to limited EEG coverage. Finally, we recently showed that learning content can be decoded in the presence of centrally recorded sleep spindles,[30,39] but decoding was based on raw EEG data rather than on spindle topographies. In short, despite converging evidence that spindle expression can be local, idiosyncratic, and flexible, we provide here the first evidence that they track participant-specific learning patterns in the service of memory consolidation. These results dovetail with a recent computational study showing that spindles promote the independent reactivation of multiple memories at network locations corresponding to awake training.[40]

It deserves mention that the spatial resolution afforded by scalp EEG is relatively coarse, only capturing macro-scale topographies of brain networks. Intracranial recordings/electrocorticography (ECoG) would provide much finer resolution, albeit at the expense of comprehensive whole-brain coverage and consistency across participants. That said, a recent study used cortical microelectrode array recordings (Utah arrays) in four pre-surgical epilepsy patients and demonstrated different spindle- and unit-firing dynamics across a 10 × 10 electrode grid (covering < 15 mm^2).[18] This suggests that spindle deployment might be sufficiently fine-tuned in space to selectively strengthen local microcircuits of learning networks.

A key open question is how exactly the deployment of spindles to learning-related cortical sites is governed. One speculative possibility is that circuit-specific encoding activation establishes transient synaptic tags. Spindles are initially broadcast widely and stochastically during sleep but resonate more strongly when coinciding with those synaptically primed circuits. This leads to higher spindle amplitudes and a concomitant increase in Ca^{2+} influx, completing the tag-and-capture cycle suggested to underlie long-term potentiation.[41] Increased spindle amplitude, as observed here and in other studies,[38,42,43] would thus reflect elevated local neural co-activation as a vestige of prior task engagement.

Another possibility is that the same thalamic circuits control the deployment of attentional resources during wake task performance and spindles during sleep. For instance, a recent study capitalized on the orientation-specific response potentiation (OSRP) in mouse primary visual cortex (V1), which reflects enhanced firing to a visual grating of particular orientation several hours after initial exposure/training. Importantly, neurons in the lateral geniculate nucleus (LGN) of the thalamus already showed orientation-selective tuning during and immediately after training. During post-training sleep, thalamocortical coherence mediated by sleep spindles drove post-sleep orientation-selective tuning in V1.[44] Additional work is needed to elucidate whether similar mechanisms apply to more complex tasks in humans, but accumulating evidence across species has linked thalamic microcircuits to a wide range of cognitive tasks.[45] This scenario, in which thalamocortical dynamics during learning bias the path of spindle deployment during sleep, is reminiscent of models of hippocampal functioning. Specifically, according to the hippocampal indexing theory,[46] the hippocampus retains pointers to cortical circuits involved in learning. Upon presentation of a partial cue, the hippocampus drives reinstatement (pattern completion) in cortical target sites. Recent work in rodents[47] and humans[48] points to a cortical-hippocampal-cortical loop around hippocampal ripples, and a tentative scenario might be that the initial cortical response in this loop is mediated by the aforementioned thalamocortical spindle projections.

Apart from spindles, memory processing during sleep has been linked to SOs and delta (1–4 Hz) rhythms, together referred to as slow wave activity (SWA).[6,19,49,50] One seminal study showed that SWA was specifically increased over central cortical areas thought to be involved in prior motor learning.[27] Likewise, wake immobilization of a participant's arm led to a decrease in SWA over corresponding motor areas.[51] Interestingly though, analogous effects were seen in the spindle/sigma band in both studies, raising the possibility that both SOs/SWA and spindles contribute to sleep-dependent consolidation. One pressing question is whether consolidation relies on concomitant or sequential occurrence of these two sleep events. Speaking of the importance of concomitant SWA spindles, a recent rodent study showed that Ca^{2+} activity was increased 3-fold when spindles were coupled to SOs.[52] The importance of coupled SO-spindle complexes has been further corroborated by a series of recent findings linking the precision of SO-spindle coupling to memory function in ageing[28,29] and to the reinstatement of prior learning experiences during sleep.[30] However, we did not observe a greater overlap of encoding patterns with spindles coupled versus not coupled to SOs in the current study (Figure S3C). Interestingly, a recent EEG/MEG study showed that the topography of spindles was unaffected by the topography of concurrent SOs.[32] This raises the possibility that the consolidation of learning patterns relies, at least in part, on the sequential occurrence of spindles and SOs. Indeed, in the original framework[15] as well as in a recent computational model,[40] it is proposed that SOs, which show enhanced prevalence during later sleep stages, further potentiate strong synapses, incidentally leading to the downscaling of weak synapses.[53] In other words, sleep-dependent consolidation might rely on a multistage tagging and capture sequence, initiated by wake task performance, potentiated by thalamocortical sleep spindles in conjunction with hippocampal ripples, and completed by SOs. Whole-night recordings would be better-suited to test this notion than the current nap design.

Apart from the nap design, there are additional limitations in the present study. First, conclusions remain correlative rather than causal. A previous study[54] used transcranial alternating current stimulation (tACS) to enhance sleep spindles, leading to improved post-sleep motor memory performance. It would be intriguing to test whether "playing back" participant-specific learning patterns in the form of exogenously induced spindle topographies enhances consolidation in our current paradigm. However, the spatiotemporal precision required for this endeavor (simultaneously inducing different spindle amplitudes at different locations across the cortex) would exceed capacities of current human non-invasive brain stimulation (NIBS) tools. Second, the nature of our paradigm precludes a direct link between encoding patterns and behavioral performance during pre-sleep retrieval. Specifically, unlike in one-shot event-related memory paradigms, retrieval performance here is the product of encoding and multiple rounds of training, including feedback. Nevertheless, the encoding pattern observed here (alpha/beta power decreases over temporo-parietal areas) is consistent with previous studies of memory formation.[55–58]

To conclude, the present study demonstrates that sleep spindles track cortical areas engaged during prior learning and that the extent of learning-spindle overlap predicts levels of memory consolidation. An exciting avenue for future work will be to elucidate the spatiotemporal dynamics between spindles, ripples, and SOs across the hippocampus and neocortical sites, both in close temporal proximity and across a whole night of sleep.

STAR★METHODS

Detailed methods are provided in the online version of this paper and include the following:

- KEY RESOURCES TABLE
- RESOURCE AVAILABILITY
  - Lead contact
  - Materials availability
  - Data and code availability
- EXPERIMENTAL MODEL AND SUBJECT DETAILS
- METHOD DETAILS
  - Memory Arena
  - Psychomotor vigilance task (PVT)
  - Procedure
  - Behavioral analysis
  - EEG data recording
  - EEG analysis: Encoding pattern
  - EEG analysis: Event detection
  - EEG analysis: Coupling of sleep spindles
  - EEG analysis: Comparison between encoding and sleep pattern
- QUANTIFICATION AND STATISTICAL ANALYSIS

SUPPLEMENTAL INFORMATION

Supplemental information can be found online at https://doi.org/10.1016/j.cub.2022.04.045.

ACKNOWLEDGMENTS

This work was supported by a Wellcome Trust/Royal Society Sir Henry Dale Fellowship (107672/Z/15/Z) and an ERC Consolidator grant (101001121) to B.P.S.

AUTHOR CONTRIBUTIONS

Conceptualization, M.P., A.C., and B.P.S.; methodology, M.P., I.C., and B.P.S.; investigation, A.C.; formal analysis, M.P. and B.P.S.; writing—original draft, M.P. and B.P.S.; writing—review & editing, M.P., A.C., and B.P.S.; visualization, M.P. and B.P.S.; supervision, G.M.B. and B.P.S.; funding acquisition, B.P.S.

DECLARATION OF INTERESTS

The authors declare no competing interests.

INCLUSION AND DIVERSITY

One or more of the authors of this paper self-identifies as a member of the LGBTQ+ community.

Received: September 1, 2021
Revised: March 11, 2022
Accepted: April 14, 2022
Published: May 12, 2022

REFERENCES

1. Diekelmann, S., and Born, J. (2010). The memory function of sleep. Nat. Rev. Neurosci. *11*, 114–126.
2. Jenkins, J.G., and Dallenbach, K.M. (1924). Obliviscence during sleep and waking. Am. J. Psychol. *35*, 605–612.
3. Müller, G.E., and Pilzecker, A. (1900). Experimentelle Beiträge zur Lehre vom Gedächtnis. Z. Psychol. *1*, 1–300.
4. Klinzing, J.G., Niethard, N., and Born, J. (2019). Mechanisms of systems memory consolidation during sleep. Nat. Neurosci. *22*, 1598–1610.
5. Lüthi, A. (2014). Sleep spindles: where they come from, what they do. Neuroscientist *20*, 243–256.
6. Born, J., and Wilhelm, I. (2012). System consolidation of memory during sleep. Psychol. Res. *76*, 192–203.
7. Fernandez, L.M.J., and Lüthi, A. (2020). Sleep spindles: mechanisms and functions. Physiol. Rev. *100*, 805–868.
8. Mednick, S.C., McDevitt, E.A., Walsh, J.K., Wamsley, E., Paulus, M., Kanady, J.C., and Drummond, S.P.A. (2013). The critical role of sleep spindles in hippocampal-dependent memory: a pharmacology study. J. Neurosci. *33*, 4494–4504.
9. Peyrache, A., and Seibt, J. (2020). A mechanism for learning with sleep spindles. Philos. Trans. R. Soc. Lond. B Biol. Sci. *375*, 20190230.
10. Gais, S., Mölle, M., Helms, K., and Born, J. (2002). Learning-dependent increases in sleep spindle density. J. Neurosci. *22*, 6830–6834.
11. Holz, J., Piosczyk, H., Feige, B., Spiegelhalder, K., Baglioni, C., Riemann, D., and Nissen, C. (2012). EEG sigma and slow-wave activity during NREM sleep correlate with overnight declarative and procedural memory consolidation. J. Sleep Res. *21*, 612–619.
12. Schabus, M., Gruber, G., Parapatics, S., Sauter, C., Klösch, G., Anderer, P., Klimesch, W., Saletu, B., and Zeitlhofer, J. (2004). Sleep spindles and their significance for declarative memory consolidation. Sleep *27*, 1479–1485.
13. Bailey, C.H., and Kandel, E.R. (1993). Structural Changes accompanying Memory Storage. Annu. Rev. Physiol. *55*, 397–426.
14. Josselyn, S.A., Köhler, S., and Frankland, P.W. (2015). Finding the engram. Nat. Rev. Neurosci. *16*, 521–534.

CellPress
OPEN ACCESS

15. Sejnowski, T.J., and Destexhe, A. (2000). Why do we sleep?—ScienceDirect. Brain Res. *886*, 208–223.
16. Rosanova, M., and Ulrich, D. (2005). Pattern-specific associative long-term potentiation induced by a sleep spindle-related spike train. J. Neurosci. *25*, 9398–9405.
17. Seibt, J., Richard, C.J., Sigl-Glöckner, J., Takahashi, N., Kaplan, D.I., Doron, G., De Limoges, D., Bocklisch, C., and Larkum, M.E. (2017). Cortical dendritic activity correlates with spindle-rich oscillations during sleep in rodents. Nat. Commun. *8*, 684.
18. Dickey, C.W., Sargsyan, A., Madsen, J.R., Eskandar, E.N., Cash, S.S., and Halgren, E. (2021). Travelling spindles create necessary conditions for spike-timing-dependent plasticity in humans. Nat. Commun. *12*, 1027.
19. Tononi, G., and Cirelli, C. (2006). Sleep function and synaptic homeostasis. Sleep Med. Rev. *10*, 49–62.
20. Petzka, M., Charest, I., Balanos, G.M., and Staresina, B.P. (2021). Does sleep-dependent consolidation favour weak memories? Cortex *134*, 65–75.
21. Hanslmayr, S., Staudigl, T., and Fellner, M.C. (2012). Oscillatory power decreases and long-term memory: the information via desynchronization hypothesis. Front. Hum. Neurosci. *6*, 74.
22. Griffiths, B.J., Parish, G., Roux, F., Michelmann, S., van der Plas, M., Kolibius, L.D., Chelvarajah, R., Rollings, D.T., Sawlani, V., Hamer, H., et al. (2019). Directional coupling of slow and fast hippocampal gamma with neocortical alpha/beta oscillations in human episodic memory. Proc. Natl. Acad. Sci. USA *116*, 21834–21842.
23. Griffiths, B.J., Martín-Buro, M.C., Staresina, B.P., Hanslmayr, S., and Staudigl, T. (2021). Alpha/beta power decreases during episodic memory formation predict the magnitude of alpha/beta power decreases during subsequent retrieval. Neuropsychologia *153*, 107755.
24. Lega, B., Germi, J., and Rugg, M. (2017). Modulation of oscillatory power and connectivity in the human posterior cingulate cortex supports the encoding and retrieval of episodic memories. J. Cogn. Neurosci. *29*, 1415–1432.
25. Noh, E., Herzmann, G., Curran, T., and de Sa, V.R. (2014). Using single-trial EEG to predict and analyze subsequent memory. Neuroimage *84*, 712–723.
26. Heib, D.P.J., Hoedlmoser, K., Anderer, P., Zeitlhofer, J., Gruber, G., Klimesch, W., and Schabus, M. (2013). Slow oscillation amplitudes and up-state lengths relate to memory improvement. PLoS One *8*, e82049.
27. Huber, R., Ghilardi, M.F., Massimini, M., and Tononi, G. (2004). Local sleep and learning. Nature *430*, 78–81.
28. Helfrich, R.F., Mander, B.A., Jagust, W.J., Knight, R.T., and Walker, M.P. (2018). Old brains come uncoupled in sleep: slow wave-spindle synchrony, brain atrophy, and forgetting. Neuron *97*, 221–230.e4.
29. Muehlroth, B.E., Sander, M.C., Fandakova, Y., Grandy, T.H., Rasch, B., Shing, Y.L., and Werkle-Bergner, M. (2019). Precise slow oscillation–spindle coupling promotes memory consolidation in younger and older adults. Sci. Rep. *9*, 1940.
30. Schreiner, T., Petzka, M., Staudigl, T., and Staresina, B.P. (2021). Endogenous memory reactivation during sleep in humans is clocked by slow oscillation-spindle complexes. Nat. Commun. *12*, 3112.
31. Mölle, M., Marshall, L., Gais, S., and Born, J. (2002). Grouping of spindle activity during slow oscillations in human non-rapid eye movement sleep. J. Neurosci. *22*, 10941–10947.
32. Klinzing, J.G., Mölle, M., Weber, F., Supp, G., Hipp, J.F., Engel, A.K., and Born, J. (2016). Spindle activity phase-locked to sleep slow oscillations. Neuroimage *134*, 607–616.
33. Nir, Y., Staba, R.J., Andrillon, T., Vyazovskiy, V.V., Cirelli, C., Fried, I., and Tononi, G. (2011). Regional slow waves and spindles in human sleep. Neuron *70*, 153–169.
34. Piantoni, G., Halgren, E., and Cash, S.S. (2017). Spatiotemporal characteristics of sleep spindles depend on cortical location. Neuroimage *146*, 236–245.
35. Cox, R., Schapiro, A.C., Manoach, D.S., and Stickgold, R. (2017). Individual differences in frequency and topography of slow and fast sleep spindles. Front. Hum. Neurosci. *11*, 433.
36. Clemens, Z., Fabó, D., and Halász, P. (2005). Overnight verbal memory retention correlates with the number of sleep spindles. Neuroscience *132*, 529–535.
37. Clemens, Z., Fabó, D., and Halász, P. (2006). Twenty-four hours retention of visuospatial memory correlates with the number of parietal sleep spindles. Neurosci. Lett. *403*, 52–56.
38. Bergmann, T.O., Mölle, M., Diedrichs, J., Born, J., and Siebner, H.R. (2012). Sleep spindle-related reactivation of category-specific cortical regions after learning face-scene associations. Neuroimage *59*, 2733–2742.
39. Cairney, S.A., Guttesen, A.Á.V., El Marj, N., and Staresina, B.P. (2018). Memory consolidation is linked to spindle-mediated information processing during sleep. Curr. Biol. *28*, 948–954.e4.
40. Wei, Y., Krishnan, G.P., Komarov, M., and Bazhenov, M. (2018). Differential roles of sleep spindles and sleep slow oscillations in memory consolidation. PLoS Comput. Biol. *14*, e1006322.
41. Redondo, R.L., and Morris, R.G.M. (2011). Making memories last: the synaptic tagging and capture hypothesis. Nat. Rev. Neurosci. *12*, 17–30.
42. Cox, R., Hofman, W.F., de Boer, M., and Talamini, L.M. (2014). Local sleep spindle modulations in relation to specific memory cues. Neuroimage *99*, 103–110.
43. Yordanova, J., Kolev, V., Bruns, E., Kirov, R., and Verleger, R. (2017). Sleep spindles in the right hemisphere support awareness of regularities and reflect pre-sleep activations. Sleep *40*, 1–13.
44. Durkin, J., Suresh, A.K., Colbath, J., Broussard, C., Wu, J., Zochowski, M., and Aton, S.J. (2017). Cortically coordinated NREM thalamocortical oscillations play an essential, instructive role in visual system plasticity. Proc. Natl. Acad. Sci. USA *114*, 10485–10490.
45. Halassa, M.M., and Kastner, S. (2017). Thalamic functions in distributed cognitive control. Nat. Neurosci. *20*, 1669–1679.
46. Teyler, T.J., and Discenna, P. (1986). The hippocampal memory indexing theory. Behav. Neurosci. *100*, 147–154.
47. Rothschild, G., Eban, E., and Frank, L.M. (2017). A cortical-hippocampal-cortical loop of information processing during memory consolidation. Nat. Neurosci. *20*, 251–259.
48. Ngo, H.V.V., Fell, J., and Staresina, B. (2020). Sleep spindles mediate hippocampal-neocortical coupling during long-duration ripples. Elife *9*, 1–18.
49. Rasch, B., and Born, J. (2013). About Sleep's role in memory. Physiol. Rev. *93*, 681–766.
50. Marshall, L., Helgadóttir, H., Mölle, M., and Born, J. (2006). Boosting slow oscillations during sleep potentiates memory. Nature *444*, 610–613.
51. Huber, R., Ghilardi, M.F., Massimini, M., Ferrarelli, F., Riedner, B.A., Peterson, M.J., and Tononi, G. (2006). Arm immobilization causes cortical plastic changes and locally decreases sleep slow wave activity. Nat. Neurosci. *9*, 1169–1176.
52. Niethard, N., Ngo, H.V.V., Ehrlich, I., and Born, J. (2018). Cortical circuit activity underlying sleep slow oscillations and spindles. Proc. Natl. Acad. Sci. USA *115*, E9220–E9229.
53. Tononi, G., and Cirelli, C. (2003). Sleep and synaptic homeostasis: a hypothesis. Brain Res. Bull. *62*, 143–150.
54. Lustenberger, C., Boyle, M.R., Alagapan, S., Mellin, J.M., Vaughn, B.V., and Fröhlich, F. (2016). Feedback-controlled transcranial alternating current stimulation reveals a functional role of sleep spindles in motor memory consolidation. Curr. Biol. *26*, 2127–2136.
55. Fellner, M.C., Bäuml, K.H.T., and Hanslmayr, S. (2013). Brain oscillatory subsequent memory effects differ in power and long-range synchronization between semantic and survival processing. Neuroimage *79*, 361–370.
56. Griffiths, B.J., Mazaheri, A., Debener, S., and Hanslmayr, S. (2016). Brain oscillations track the formation of episodic memories in the real world. Neuroimage *143*, 256–266.

57. Hanslmayr, S., Spitzer, B., and Bäuml, K.H. (2009). Brain oscillations dissociate between semantic and nonsemantic encoding of episodic memories. Cereb. Cortex *19*, 1631–1640.

58. Klimesch, W., Schimke, H., Doppelmayr, M., Ripper, B., Schwaiger, J., and Pfurtscheller, G. (1996). Event-related desynchronization (ERD) and the Dm effect: does alpha desynchronization during encoding predict later recall performance? Int. J. Psychophysiol. *24*, 47–60.

59. The Mathworks Inc. MATLAB and statistics toolbox. https://www.mathworks.com/products/matlab.html.

60. Brainard, D.H. (1997). The psychophysics toolbox. Spat. Vis. *10*, 433–436.

61. Mehrer, J., Spoerer, C.J., Jones, E.C., Kriegeskorte, N., and Kietzmann, T.C. (2021). An ecologically motivated image dataset for deep learning yields better models of human vision. Proc. Natl. Acad. Sci. USA *118*, 1–9.

62. Kriegeskorte, N., Mur, M., Ruff, D.A., Kiani, R., Bodurka, J., Esteky, H., Tanaka, K., and Bandettini, P.A. (2008). Matching categorical object representations in inferior temporal cortex of man and monkey. Neuron *60*, 1126–1141.

63. Oostenveld, R., Fries, P., Maris, E., and Schoffelen, J.M. (2011). FieldTrip: open source software for advanced analysis of MEG, EEG, and invasive electrophysiological data. Comput. Intell. Neurosci. *2011*, 156869.

64. Berens, P. (2009). CircStat: a MATLAB toolbox for circular statistics. J. Stat. Softw. *31*, 1–21.

65. Maris, E., and Oostenveld, R. (2007). Nonparametric statistical testing of EEG- and MEG-data. J. Neurosci. Methods *164*, 177–190.

66. Staresina, B.P., Bergmann, T.O., Bonnefond, M., van der Meij, R., Jensen, O., Deuker, L., Elger, C.E., Axmacher, N., and Fell, J. (2015). Hierarchical nesting of slow oscillations, spindles and ripples in the human hippocampus during sleep. Nat. Neurosci. *18*, 1679–1686.

67. Ngo, H.V.V., Martinetz, T., Born, J., and Mölle, M. (2013). Auditory closed-loop stimulation of the sleep slow oscillation enhances memory. Neuron *78*, 545–553.

68. Holm, S. (1979). A simple sequentially rejective multiple test procedure. Scand. J. Stat. *6*, 65–70.

69. R Core Team (2013). R: A Language and Environment for Statistical Computing (R Foundation for Statistical Computing).

70. Allen, M., Poggiali, D., Whitaker, K., Marshall, T.R., and Kievit, R.A. (2019). Raincloud plots: A multi-platform tool for robust data visualization (version 1; peer review: 2 approved). Wellcome Open Res. *4*, 63.

STAR★METHODS

KEY RESOURCES TABLE

REAGENT or RESOURCE	SOURCE	IDENTIFIER
Deposited data		
OSF	https://osf.io/a3phq/	a3phq
Software and algorithms		
MATLAB	https://uk.mathworks.com/products/matlab.html	2016a
Psychophysics Toolbox	http://psychtoolbox.org	3.0.14
FieldTrip Toolbox	www.fieldtriptoolbox.org	v.06/02/2017
CircStat Toolbox	https://www.jstatsoft.org/article/view/v031i10	N/A
Raincloud plots	https://github.com/RainCloudPlots/RainCloudPlots	N/A
R	https://www.r-project.org	3.4.3
Other		
Brain Products	http://www.brainproducts.uk	EEG System

RESOURCE AVAILABILITY

Lead contact

For further information or requests, contact should be directed towards the lead contact, Bernhard Staresina (bernhard.staresina@psy.ox.ac.uk).

Materials availability

This study did not generate new, unique reagents or materials.

Data and code availability

- De-identified and pre-processed data have been deposited at the open science framework (OSF: https://osf.io/a3phq/). They are publicly available as of the date of publication.
- All original code has been deposited at the open science framework (OSF: https://osf.io/a3phq/) and is publicly available as of the date of publication.
- Any additional information required to reanalyse the data reported in this paper is available from the lead contact upon request.

EXPERIMENTAL MODEL AND SUBJECT DETAILS

22 participants were tested. Due to technical issues during data collection, 3 participants had to be excluded resulting in 19 participants for the final sample ($mean_{age}$ = 20.7, $range_{age}$ = 18-31, female = 15).

Pre-screening ensured that participants had no history of neurological or psychiatric disorders and a normal sleep-wake cycle. Participants were instructed to get up one hour earlier than normal and avoid caffeine the day of the experiment. After participating in the study, participants received monetary reimbursement. The study was approved by the University of Birmingham Research Ethics and Governance Committee and written informed consent was obtained from all participants before the start of the study.

METHOD DETAILS

Memory Arena and PVT were implemented via custom scripts in MATLAB 2016a (MathWorks, Natick, USA).[59] For the PVT, functions of the Psychophysics Toolbox Version 3.0.14[60] were additionally used.

Memory Arena

The *Memory Arena* consists of a circle divided into coloured quarters (upper left: blue, upper right: green, lower right: yellow, lower left: red). Within the circle, objects are sequentially presented in different spatial positions. Participants have to learn both the sequence in which the objects were presented as well as the spatial position of each object.

20 target objects (images of 5 faces, 5 natural objects, 5 animals and 5 manmade objects) were randomly selected from a stimulus pool of 40 objects (coloured and presented on a grey 90x90 pixels square).[61,62] The spatial position of each object was restricted by

the position of other objects. Consequently, there was no overlap between objects, but objects possibly covered more than one colour wedge.

Psychomotor vigilance task (PVT)

A white fixation cross was presented in the middle of the screen. After on average 12.5 seconds (jitter ± 2.5s), a counter replaced the fixation cross. The counter started at 0 and counted forward in 20 ms steps to 2000. Upon start of the counter, participants had to press the space bar as fast as possible. After the key press, feedback about their reaction time was displayed for 2s (Figure 1B). Overall, the PVT lasted ~5 minutes (19 trials on average, approximately matching the number of motor responses deployed during the MemoryArena encoding session).

Procedure

The experimental session started at 10 am with the application of electroencephalography (EEG), electromyography (EMG) and electrooculography (EOG).

Approximately one hour later at 11 am, participants started with the first PVT, which was preceded by a short practice session (~20 sec). Before they continued with the Memory Arena, participants received written instructions and performed a practice session (with 3 objects) of each Memory Arena part (encoding, training and retrieval). Participants were instructed to associate and combine the objects into a coherent story.

During the encoding part of the Memory Arena, all 20 objects were sequentially presented within the circle. Participants confirmed processing of each object by clicking on it. The current object then disappeared, and the next object was presented.

Directly after the encoding part, a training session was conducted. The training session started with all 20 objects arranged around the arena. The objects had to be dragged and dropped in the correct sequence to their correct spatial position. If an error was made regarding the sequence or spatial position, the arena turned red and the error was corrected. Sequence errors were defined as object i not being placed at the ith position. Spatial errors were defined as the overlap between correct and chosen position of an object being less than 25%. After potential error corrections, the object remained at its correct spatial position and the next object had to be selected and placed in the arena. When all 20 objects were placed, feedback about the overall performance was presented. The overall performance was defined as the number of correct objects divided by the total number of objects, where an object was classified as correct when sequence as well as spatial position were correct. Participants finished training after reaching 70% overall performance in two consecutive runs.

A second PVT then followed the training session. After the PVT, pre-sleep retrieval was assessed. Like the training session, retrieval started with all 20 objects arranged around the arena which had to be dragged and dropped in the correct sequence to their correct spatial position. Importantly though, errors were not corrected and no feedback was provided.

Participants started the 2-hour nap between 1pm - 2.30pm (see Table S1 for descriptive sleep data). Following the nap, participants continued with an interference task. The purpose of the interference task was to increase sensitivity to sleep-dependent consolidation effects. In a previous study, we used the same task (Memory Arena) and found sleep-dependent consolidation effects with a combination of a 2x70% training threshold (70% overall performance in two consecutive runs) and the induction of retroactive interference directly after sleep.[20] Therefore, we applied the same methods in this study.

During the interference task, participants had to encode the same 20 objects but in a different sequence and at different spatial positions (Figure 1A). The difference between the old and the new, interfering, spatial position of every object was at least 5 pixels (Euclidean distance). Encoding of the interfering positions was conducted in the same way as the original encoding. Subsequently, participants had to retrieve the interfering sequence and spatial positions (without prior training). Lastly, another PVT and the post-sleep retrieval (of the original sequence and spatial positions) were performed. Until the start of the post-sleep retrieval test, participants were unaware of the final test.

Behavioral analysis

In the behavioral precursor to this study, we found that sequence performance was the most sensitive measure to capture sleep-dependent memory consolidation.[20] Consequently, all following analyses focus on sequence performance.

To calculate sequence performance, the selected order of all 20 object was correlated with a vector ranging, in ascending order, from 1 to 20. This correlation approach is preferable to simply counting the correct sequence position of every object, as it reflects both the correct sequence position of every object as well as correct transitions between objects. Correlation values were Fisher z-transformed for further statistical analyses. To test for a significant sequence performance change from pre- to post-sleep retrieval, a paired t-test was computed.

For correlating the change in sequence performance with the EEG data, a sequence retention score was calculated as the relative change from pre- to post- sleep sequence retrieval: 100*(post/pre).

EEG data recording

EEG data were recorded using a Brain Products 64-channel EEG system and were sampled at a rate of 1000 Hz. Electrodes were arranged according to the 10-20 system (including FCz as reference, AFz as ground and left and right mastoids). Two electrodes were placed on the chin to record muscle activity (electromyography, EMG) and two electrodes recorded eye movements (electrooculography, EOG), leaving 58 channels to record scalp EEG.

EEG analysis: Encoding pattern

EEG analyses were performed using the FieldTrip toolbox,[63] CircStat Toolbox[64] and custom written scripts in MATLAB.

To remove eye movements from the data, an independent component analysis (ICA) was used. Data were down-sampled to 200 Hz (including an anti-aliasing filter implemented in the MATLAB built-in function *resample*), re-referenced to linked mastoids, filtered (high-pass: 1 Hz, low-pass: 100 Hz, band-stop filter: 48-52 Hz), demeaned and segmented in 2 second epochs for a first visual artifact rejection. All phases of the Memory Arena and the PVT were concatenated, coarse artifacts were removed based on outliers regarding amplitude, kurtosis and variance (implemented in *ft_rejectvisual*) and bad channels were rejected. Based on those data, the unmixing matrix was obtained and bad components were identified. The raw data were then preprocessed again, as the first pre-processing was optimized for ICA. The data were down-sampled to 200 Hz (including an anti-aliasing filter), re-referenced to linked mastoids, filtered (high-pass: 0.3 Hz, low-pass: 40 Hz) and demeaned. The unmixing matrix was applied to the new preprocessed data and bad channels were interpolated.

To derive encoding patterns, the encoding part of the Memory Arena was contrasted against the first PVT. Note that the first PVT was conducted in close temporal proximity to the Memory Arena and requires sustained attention but no memory-related processes. The data recorded during the encoding part of the Memory Arena and the PVT were segmented into 1 second epochs (50% overlap), tapered with a Hanning window and transformed from time to frequency domain using Fast Fourier Transformation. To facilitate reproducibility of results, artifacts were defined, on each channel, based on the 95th percentile uniquely for each frequency bin distribution (across epochs). Within each participant, all 1 second epochs above the 95th percentile were labelled as artifacts and excluded. Note that results did not qualitatively change as a function of the chosen exclusion criterion (applying the 90th or 85th percentile or the mean + 2.5 SD as a threshold revealed similar results, see Figure S1E) or when muscle artifacts were excluded based on channel-wise visual inspection (see Figures S1D and S1E, averaged across participants 2% of epochs (absolute: 14) were excluded). Power spectra obtained from encoding and PVT were contrasted (encoding - PVT), yielding absolute power changes during encoding relative to the PVT.

Significant frequency bins were defined based on the group statistics by applying a two-sided cluster-based permutation test with 1000 randomisations.[65] The topography for each participant was then derived by collapsing power values across the significant frequency bins for each channel separately resulting in a 1 x channel (=58) vector.

EEG analysis: Event detection

Sleep spindles and SOs were detected for each participant, based on established detection algorithms.[66,67] Like wake data, sleep data were down-sampled to 200 Hz (including an anti-aliasing filter), re-referenced to linked mastoids and filtered (high-pass: 0.3 Hz, low-pass: 40 Hz). Bad channels were matched between wake and sleep data, excluded and interpolated. Finally, to identify and mark coarse artifacts, data were visually inspected. Channelwise event detection of both sleep spindles and slow oscillations were conducted on data from non-rapid eye movement (NREM) sleep stages 2 and 3. Events were only included if free of artifacts between 1s before and 1s after the event. Note that the results did not significantly differ between sleep stage 2 and 3 (data not shown).

To detect fast sleep spindles, data were band-pass filtered between 12-15 Hz (4th order two-pass Butterworth filter). The envelope was calculated based on the smoothed signal (200ms moving average) using the Hilbert transform. An amplitude criterion (mean + 1.25*SD) was applied to the signal. Sleep spindles were detected when the signal exceeded the amplitude criterion for more than 0.5 but less than 3 seconds (duration criterion).

The maximum of the envelope of each detected spindle was used as the amplitude measure. Duration was the time from beginning to end of each event and density was calculated as the number of detected events / total (artifact free) time spent in NREM sleep stage 2 and 3.

To detect slow oscillations, data were band-pass filtered between 0.3-1.25 Hz (4th order two-pass Butterworth filter). Zero crossings were identified, and three criteria (duration criterion, trough to peak criterion and amplitude criterion) had to be fulfilled. The length criterion was met if one positive to negative crossing was followed by a second positive to negative crossing within a time window of 0.8 to 2 seconds. Based on all sufficiently long events, mean and standard deviation were calculated for trough to peak amplitudes as well as for absolute values of trough amplitudes. All events exceeding both means + 1.25*SDs were considered slow oscillations.

The amplitude of SOs was defined as the most negative trough (downstate). To facilitate comparability between sleep measures, the absolute value of SO amplitudes was used. Thus, the downstate became positively scaled to match all other sleep measures. Duration was defined as the time between the first positive to negative and the following positive to negative crossing. Slow oscillation density was calculated by dividing the number of detected events by the total (artifact free) time spent in NREM sleep stages 2 and 3.

Amplitude, duration and density of detected spindles and SOs were extracted per channel and averaged across events (amplitude, duration) resulting in 6 different 1 x channel (=58) vectors.

EEG analysis: Coupling of sleep spindles

For each spindle event, the phase of the EEG trace filtered in the SO frequency band was extracted. To this end, the data around each spindle event were filtered from 0.3 – 1.25 Hz. After applying a Hilbert transform, the instantaneous phase angle at the maximum of the envelope of each detected spindle was extracted.

To compare spindles with higher vs. lower coupling, all spindle events per channel were classified based on their phase value. The 50% of spindle events with a phase value closest to 0 degrees were classified as spindles with a higher coupling. The remaining 50% of spindle events were classified as spindles with a lower coupling.

EEG analysis: Comparison between encoding and sleep pattern

For each participant, the 1x58 vector obtained from encoding was correlated (Spearman's rho) with every 1x58 vector of the sleep characteristics (amplitude, duration and density for spindles and SOs). Correlations were then Fisher z-transformed for group statistics.

QUANTIFICATION AND STATISTICAL ANALYSIS

For statistical significance an alpha level of 0.05 was used as a threshold. Correlation distributions between encoding and sleep topographies were tested with a 2 (event type: spindles vs. SOs) x 3 (event characteristic: amplitude, duration, density) repeated measures ANOVA. Paired sampled t-tests and one-sample t-tests were used for post-hoc comparisons. Post-hoc tests were corrected using the Bonferroni-Holm correction.[68] For effect sizes, we report partial eta squared (η_p^2) for the ANOVA and Cohen's d for t-tests.

Partial correlations were conducted to test for mediating effects of spindle characteristics (e.g., spindle density) on the correlation between encoding and another spindle characteristic (e.g., spindle amplitude).

To test for an association between sequence retention and the encoding-spindle overlap, the Spearman's rho correlation was conducted. To rule out that the correlation with behaviour is solely driven by either sleep spindles or by encoding power, we applied a permutation approach and shuffled topographies between participants (1000 permutations). To obtain the observed correlation, we derived, for each participant, (i) behavioural performance, (ii) encoding topography and (iii) sleep spindle topography. By shuffling only one topography (encoding or sleep spindles) between participants while retaining the other participant-specific topography and behavioural performance, two null distributions were generated: First, a distribution under the null hypothesis that the participant-specific encoding topography is irrelevant for the correlation with behaviour (shuffling the encoding topographies). Second, a distribution under the null hypothesis that the participant-specific sleep spindle topography is irrelevant for the correlation with behaviour (shuffling the spindle topographies). The observed correlation was then tested against both null distributions.

All statistical details are available in the results section and/or figure legends. Statistical testing was performed in MATLAB and R (Version 3.4.3)[69] and for data visualisation raincloud plots were used.[70]

Current Biology, Volume *32*

Supplemental Information

Sleep spindles track cortical learning patterns for memory consolidation

Marit Petzka, Alex Chatburn, Ian Charest, George M. Balanos, and Bernhard P. Staresina

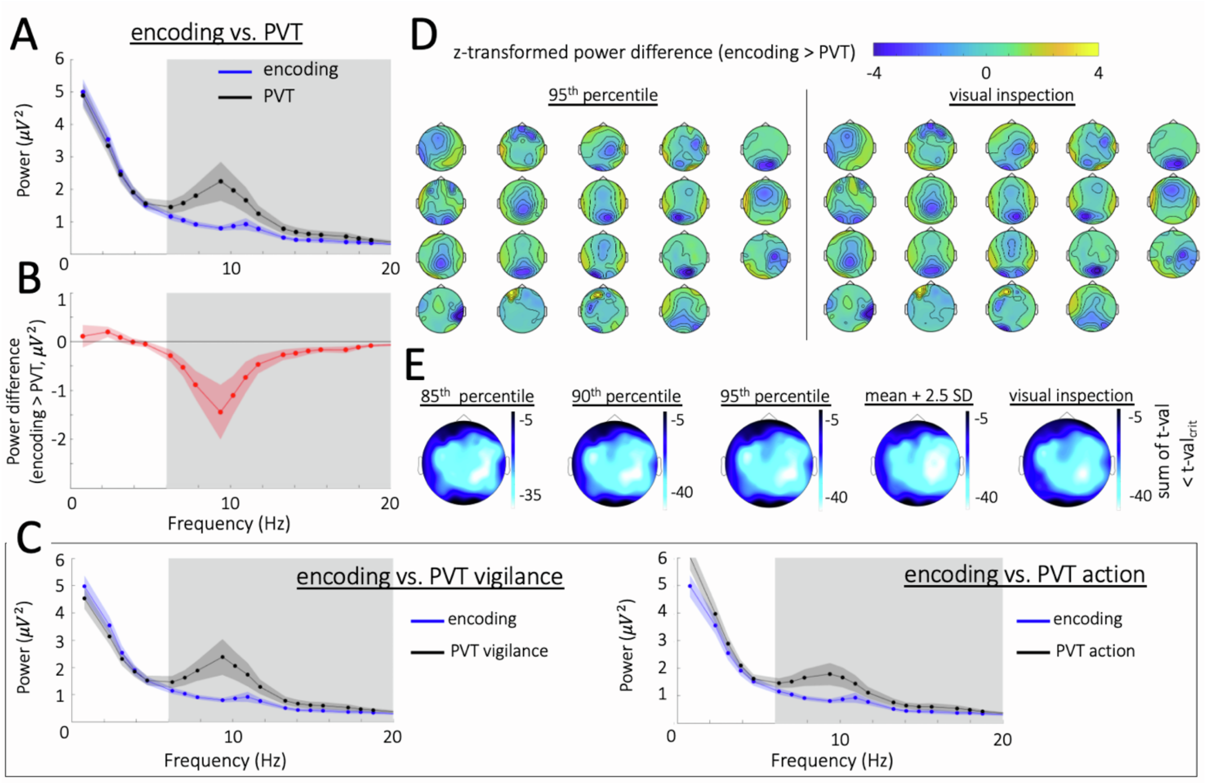

Figure S1. Frequency spectra and topographies of power differences between encoding and PVT. Related to Figure 1E.

(A) Power spectra (mean +/- SEM) for encoding (blue), PVT (black) as well as their difference (red, shown in B, statistically significant differences are highlighted in grey, $p < .05$ cluster corrected) are shown for electrode CP4 (cf., main Figure 1E). These were the raw values entering statistical analyses presented in main Figure 1E. (C) Power spectra (mean +/- SEM) for encoding and PVT 'vigilance' (left) as well as encoding and PVT 'action' (right). For the PVT 'vigilance' component, 10 sec pre-counter (minimum for which the fixation cross, without any motor responses, was presented) and for the PVT 'action', 4 seconds post-counter (time during which motor responses were given) were included. Statistically significant differences are highlighted in grey, $p < .05$ cluster corrected. (D) Participant-specific topographies of the 6-20 Hz power changes during encoding relative to the PVT (after excluding epochs based on the 95th percentile or after visual inspection). Power changes were z-transformed for comparability across participants. (E) Applying different thresholds (85th, 90th, 95th percentile, mean + 2.5 SD or visual inspection) to exclude potential artifacts result in comparable encoding patterns (the frequency range in which encoding and PVT significantly differ is always 6-20Hz).

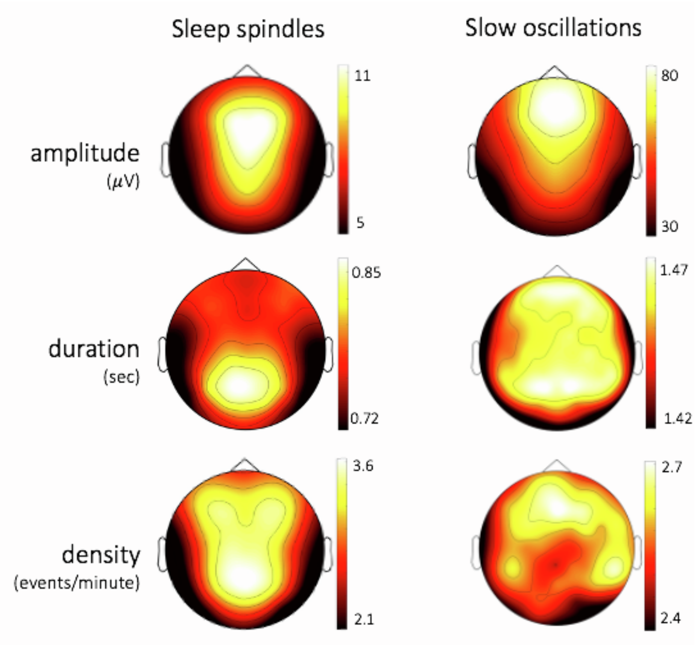

Figure S2. Group-level topographies of amplitude, duration and density of sleep spindles and slow oscillations. Related to Figure 2A.

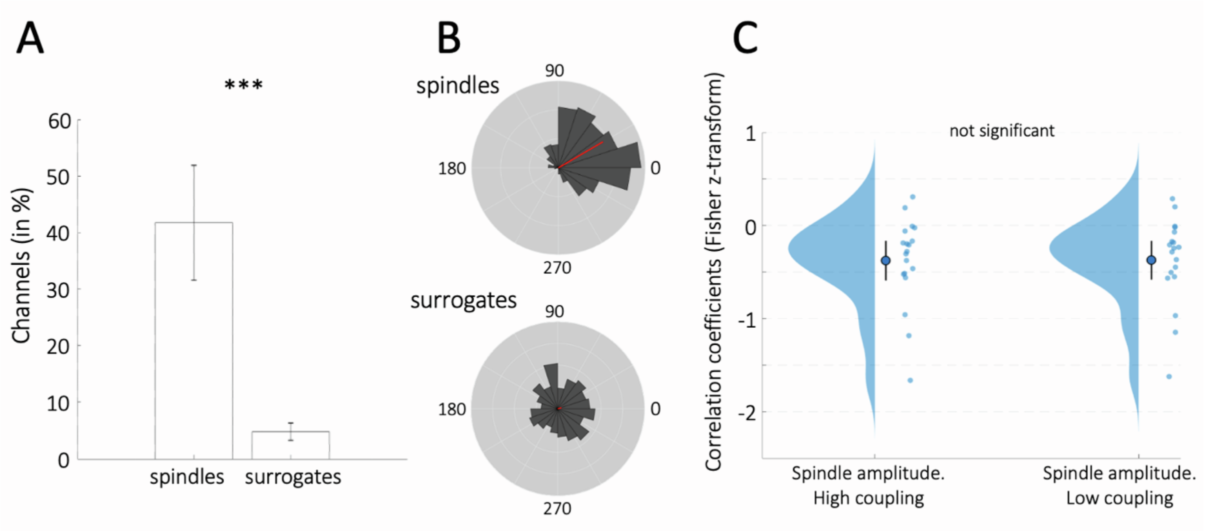

Figure S3. Coupling of spindles and surrogate events to the phase of the signal filtered in the SO frequency range (0.3 -1.25). Related to Figure 2B.

(A) Mean (+/- 95% confidence intervals) percentage of channels on which sleep spindles and surrogate events are significantly coupled (defined by a significant deviation from a uniform distribution, Rayleigh test: $p < .05$). Spindles are coupled to SOs on significantly more channels than surrogates. Surrogates were matched control events - for each detected spindle, a spindle-free epoch within 15 seconds before or after the actual spindle event was identified [S1]. The instantaneous phase angle of the SO filtered and Hilbert transformed signal was then extracted at the centre of the spindle-free epoch.

(B) The corresponding phase (in degrees) of spindle maxima (top) and surrogate centres (bottom) plotted across all channels with significant spindle coupling (including all participants, fixed-effects, $n = 447$). While spindles significantly cluster at a phase of 30 degrees (Rayleigh test: $z = 173.75$, $p < .001$, resultant vector length = 0.62), surrogates deviate only moderately from a uniform distribution (Rayleigh test: $z = 3.22$, $p = .040$, resultant vector length = 0.08). Both distributions significantly differ (Kuiper test: $p < .001$).

(C) No differential encoding-spindle amplitude overlap for spindles with higher (left) vs. lower (right) coupling to the SO up-state.

	N1	N2	N3	REM	TST
mean	18.39	52.53	10.42	16.24	103.58
SEM	2.46	3.66	2.56	2.85	2.28

Table S1. **Descriptive sleep data in minutes (mean and SEM). Related to Figure 1C and 2A.**
n = 19. TST = total sleep time.

**electrode Fz**	**sleep spindles**			**slow oscillations**		
	amplitude	duration	density	amplitude	duration	density
mean	11.13	0.76	3.27	82.97	1.42	2.49
SEM	0.63	0.01	0.16	6.11	0.01	0.13
**electrode Cz**						
mean	11.03	0.80	3.45	68.07	1.41	2.67
SEM	0.54	0.01	0.19	4.49	0.01	0.12
**all electrodes**						
mean	7.83	0.78	3.02	56.01	1.42	2.62
sem	0.36	0.01	0.15	4.14	0.01	0.12

Table S2. **Descriptive data of sleep oscillations. Related to Figure 2A.**
Descriptive data of amplitude (maximum peak for spindles and absolute value of the most negative trough for slow oscillations, in μV), duration (time from beginning to end of algorithmically detected events, in seconds) and density (n events/minute) of sleep spindles and slow oscillations for electrode position Fz, Cz and averaged across all electrodes (mean and SEM). n = 19.

Supplemental References

S1. Ngo, H.V. V., Fell, J., and Staresina, B. (2020). Sleep spindles mediate hippocampal-neocortical coupling during long-duration ripples. Elife *9*, 1–18.
